# Assessing COVID-19 lockdown effects on coastal water quality in a strongly impacted tourist destination using Sentinel-2 multispectral data

**DOI:** 10.1371/journal.pone.0334974

**Published:** 2025-10-30

**Authors:** Francisco Flores-de-Santiago, Felipe Amezcua, Ranulfo Rodríguez-Sobreyra, León Felipe Álvarez-Sánchez, Luis Valderrama-Landeros, Francisco Flores-Verdugo

**Affiliations:** 1 Instituto de Ciencias del Mar y Limnología, Unidad Académica Procesos Oceánicos y Costeros, Universidad Nacional Autónoma de México, Coyoacán, Ciudad de México, Mexico; 2 Instituto de Ciencias del Mar y Limnología, Unidad Académica Mazatlán, Universidad Nacional Autónoma de México, Mazatlán, Sinaloa, Mexico; 3 Instituto de Ciencias del Mar y Limnología, Unidad de Informática Marina, Universidad Nacional Autónoma de México, Coyoacán, Ciudad de México, Mexico,; 4 Coordinación de Geomática, Comisión Nacional Para el Conocimiento y Uso de la Biodiversidad, Tlalpan, Ciudad de México, Mexico; Amity University Amity Institute of Biotechnology, INDIA

## Abstract

Remote sensing data from satellite platforms were the only available source of information for environmental studies during the COVID-19 lockdown in many regions of the world. We analyzed the spatial variability of representative water indices derived from the Sentinel-2 sensor across six coastal land cover classes along a tourist destination on the North Pacific coast of Mexico. A comparative assessment was conducted between the 2020 lockdown period and the same holiday season in 2019, 2020, and 2022, evaluating the spatial distribution of water indices per coastal class. Principal coordinate analysis of organic content matter (CDOM), Chlorophyll-a (CHLA), and total suspended matter (TSMC2 and TSM_Clear) indices demonstrated clear distinctions in water quality among pre-pandemic (2019), pandemic (2020), and post-pandemic (2021−2022) periods. Canonical analysis of principal coordinates during the lockdown year revealed two key patterns: (1) sewage and harbor areas displayed a significant decrease in CHLA levels alongside elevated TSMC2, while (2) mangrove forest exhibited markedly reduced CDOM in post-pandemic years. Distance-based redundancy analysis further showed interannual variability across coastal zones, while the pandemic year (2020) was particularly distinguished by diminished CDOM in tourist and industrial areas. The high-resolution (10 m/pixel) and revisit time (5 days) of Sentinel-2 data was invaluable for monitoring water quality dynamics during the COVID-19 lockdown.

## Introduction

Coastal ecosystems—including sandy beaches, coral reefs, and mangrove forests—face increasing degradation from urban expansion and tourism development [1]. Tourist activity degrades coastal water quality through two primary pathways: (i) sewage discharge and sediment resuspension increase total suspended matter (TSM), reducing light penetration [2], and (ii) terrestrial runoff elevates colored dissolved organic matter (CDOM), reflecting sediments, dissolved solids [3], and organic pollution [4]. These optical water quality parameters (TSM, CDOM) serve as critical indicators of ecosystem stress, impairing primary productivity in phytoplankton and seagrass communities while altering water clarity [5]. Thus, systematic monitoring of these variables enables discrimination between natural variability and anthropogenic impacts, providing a scientific basis for targeted conservative strategies [6].

Contemporary water quality monitoring utilizes complementary methodologies: (i) direct measurements through in situ sensors (conductivity-temperature-depth probes, fluorometers) and laboratory analyses (UV-visible spectrophotometry), and (ii) indirect observations via orbital spaceborne platforms. Although in situ techniques deliver accurate, high-resolution chemical data (typically at µg/L detection limits), their deployment is often constrained by logistical and infrastructural limitations, particularly in regions without historical baseline monitoring infrastructure [7]. In contrast, satellite-based sensors offer unparalleled spatiotemporal coverage, with data routinely archived in global open-source repositories [8]. Numerous studies have validated the capability of remote sensing to quantify parameters [9]—including TSM, CDOM, and chlorophyll-a (CHLA, an ecological indicator of phytoplankton response to nutrient enrichment)—across diverse aquatic systems, from coastal lagoons [10] and lakes [6] to rivers [11], particularly where in situ data are unavailable.

In January 2020, the World Health Organization declared the COVID-19 (novel coronavirus SARS-CoV-2) outbreak a global health emergency, which was later classified as a pandemic in March of the same year. As a result, countries worldwide implemented various measures, including travel restrictions and lockdowns, to contain the spread of the virus [12]. Mexico´s response followed a characteristic timeline: after confirming its first COVID-19 case on February 28, 2020, the government instituted nationwide mobility restrictions effective March 23—mirroring the 20–25 day response window observed in other mid-latitude nations. These extraordinary circumstances created a natural experiment for environmental scientists, as traditional field monitoring became largely infeasible during peak restriction periods. Satellite remote sensing emerged as the principal methodological approach for environmental assessment during this interval. Peer-reviewed studies have established pandemic-related environmental changes across multiple systems: atmospheric particulate reduction across major cities [13–15], inland water quality improvements [2,16], and ecosystem recovery indicators [17]. Therefore, this study aimed to evaluate the impact of lockdown measures on the coastal waters of Mazatlan, Mexico, using data from the Sentinel-2 mission. We expect to observe changes in the content of TSM and other variables due to the lack of tourist activities during the lockdown.

## Materials and methods

### Study area

Mexico ranks among the world´s most visited nations, receiving approximately 45 million international tourists annually. The tourist sector accounts for 9% (USD 14.7 billion) of the national GDP in 2019, with sun and beach tourism dominating this revenue stream [18]. This economic significance stems from two key assets: (i) 10,545 km of coastline spanning the Pacific, Gulf of California, and Caribbean Sea [19], and (ii) 35 UNESCO-designated cultural heritage sites.

**Fig 1:**
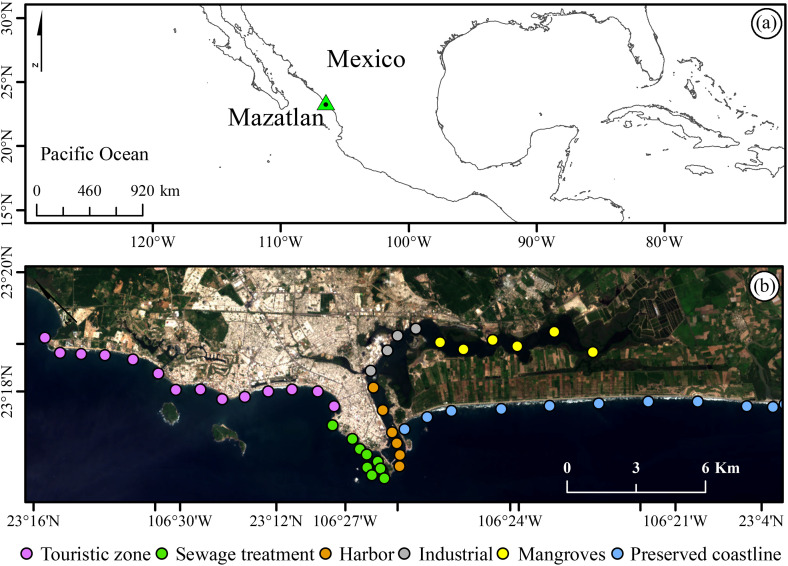
Map of the northwestern region of Mexico showing the location of the city of Mazatlan (a). The small colored circles indicate the position and class of the extracted Sentinel-2 pixels **(a)**. The coastline of Mexico was acquired from the Natural Earth website–(http://www.naturalearthdata.com/about/terms-of-use/). Landsat-8 image courtesy of the **U.**S. Geological Survey **(b)**.

Mazatlan stands as one of Mexico´s coastal getaways, drawing travelers with its vibrant cultural traditions and recreational sandy beaches. Situated along the Pacific coastline at the entrance of the Gulf of California, the city experiences a distinctive hot semi-arid climate (Köppen BSh classification), marked by temperatures averaging 24–28 °C year-round (Fig 1), while annual rainfall varies considerably across the region from 900 to 1300 mm [20]. Mazatlan´s coastline features gently sloping shores with semi-diurnal tidal patterns, where summer spring tides (June–September) can surge to 1.8 m [21]. The city´s prized 24-km beachfront, while a major tourist asset, faces growing threats from erosion that jeopardize coastal infrastructure in vulnerable zones [22]. Mazatlan´s international airport offers direct flights to key North American markets, while its bustling harbor welcomes dozens of cruise ship visits each year—including regular calls from industrial giants like Norwegian, Princess, Royal Caribbean, and Carnival [23]. However, the city´s primary wastewater treatment facilities operate beyond capacity—one pumping minimally processed effluent through an offshore pipeline at the harbor entrance, while another directs flow into the industrial district. Additionally, clandestine pipelines contribute to untreated waste disposal in the harbor, exacerbating contamination in the industrial area [24].

### COVID-19 and Tourist arrivals data

Our study period spanned from January 2019 to February 2023 for pandemic metrics and January 2017 through December 2022 for tourism data. We tracked weekly COVID-19 cases and fatalities using the Johns Hopkins University Center for Systems Science and Engineering dashboard (https://gisanddata.maps.arcgis.com/apps/dashboards/bda7594740fd40299423467b48e9ecf6). Tourism statistics, including both domestic and international visitor numbers, were obtained directly from Mexico´s Secretary of Tourism (Secretaría de Turismo) official records (https://www.datatur.sectur.gob.mx/SitePages/ActividadHotelera.aspx).

### Satellite data and water indices

We established 48 water monitoring stations approximately 100 m from the water´s edge to reduce surf zone interference, a distance based on the beach slope characteristics of these collision coasts [22]. The stations are classified into six distinct categories based on the environmental and industrial conditions characteristic of the Mazatlán coast (Fig 1). For satellite data, we utilized the Sentinel-2 multispectral sensor, which captures surface reflectance across 13 spectral bands at spatial resolutions of 10–60 m—particularly valuable for monitoring internal coastal environments like estuaries, bays [25], and coastal lagoons [8].

From the Sentinel-2 archive, we extracted four key bands (B3: green; B4: red; B5: red edge 1; and B7: red edge 3 for all available cloud-free acquisitions across four distinct periods between April and July: pre-lockdown (2019), peak lockdown (2020), and two post-pandemic years (2021–2022). All the time series data and processing were conducted in Google Earth Engine (GEE), integrated within Quantum GIS v.3.22.8, where we systematically evaluated four water quality indices (Table 1) for environmental assessment. When in situ measurements are unavailable for empirical model calibration, these bio-optical algorithms offer a robust and reproducible method for estimating key water quality parameters—including CHLA, CDOM, and TSM [26]. Such algorithms have been particularly well-suited for optically complex waters, such as coastal zones, estuaries, and lacustrine environments [27].

**Table 1:** Algorithms for the water indices used in this study. B3 (green), B4 (red), B5 (red edge 1), B7 (red edge 3).

Water index	Algorithm	Reference
TSM Clearwater	TSM_(mg/L)_ = 4.044 x e^(19.53 x B4)	[6]
CDOM	CDOM_(m-1)_ = 6.68 x (B3/ B4)^-3.12	[11]
Chlorophyll‒a	Chla_(ug/L)_ = 14.039 + 86.115 x (B5 – B4/ B5 + B4) + 194.325 x (B5 - B4/ B5 + B4)^2	[11]
TSM Case 2 water	C2RCC‒Nets	[28]

### Statistical analysis

We determined the pandemic´s temporal impacts by analyzing 2020 tourist arrivals at Mazatlan´s port to establish distinct study periods between April and July: pre-pandemic (2019), peak lockdown (2020), and post-lockdown recovery (2021–2022). For each period, we processed time-series data from all sampling stations, calculating descriptive statistics (mean and standard deviation) for the water indices (as listed in Table 1) across six distinct coastal classes (Fig 1). This stratified approach allowed us to evaluate both temporal trends and spatial variability in water quality parameters.

We assess spatiotemporal environmental patterns (year and coastal class) by conducting multivariate analyses. To do this, an environmental matrix containing *j* columns (stations) and *i* lines (environmental factors) was elaborated, and the corresponding Euclidean distance similarity matrices were generated. All variables were normalized to a mean of 0 and a standard deviation of 1 to account for measurement scale differences.

With the similarity matrix, we assessed temporal differences across every coastal class through hierarchical multivariate analyses using year and coastal class as categorical factors, and a two-way permutational multivariate analysis of variance (PERMANOVA) was employed to test the null hypothesis (H_0_) that no significant differences existed among pre-pandemic (2019), pandemic (2020), and post-pandemic (2021–2022) periods in the different coastal classes across all measured environmental parameters (p < 0.05 significant threshold). Type I sums of squares (fixed effects) and type III significance testing using 10,000 unrestricted raw data permutations were used, complemented by PERMDISP analysis to verify dispersion homogeneity across periods. Significant effects were further examined through pairwise comparisons with Bonferroni correction by adjusting the significance level (α = 0.05) through division by the total number, to correct multiple comparisons.

In case significant results existed, different graphical analyses were performed to visually show these differences. Thus, a Canonical Analysis of Principal Coordinates (CAP) was performed to visualize spatial and temporal clustering patterns among sampling stations based on environmental factors. This ordination technique generates a two-dimensional scatterplot showing: (i) station grouping by zone and year, and (ii) overlaid environmental vectors indicating the direction and strength of each factor´s influence. The vector overlay in our CAP ordination was generated using Pearson correlations, specifically highlighting linear relationships between the variables and the CAP axes. These revealed key environmental drivers of spatial and temporal patterns, where vector direction indicated the nature of each parameter´s influence while vector magnitude reflected its discriminatory power. The biplot axes (range: -n to n) positioned sampling stations relative to this environmental gradient, with the centroid of value 0,0 marking the theoretical null position where no between-group differences would occur if H_0_ were true. Notably, vectors extending farther from the centroid identified the most influential parameters shaping the observed cluster separation.

We employed a distance-based linear model permutation test (DistLM), meeting the assumption of this analysis (the number of samples was higher than the number of variables). In this analysis, the variation in the data is partitioned and displayed as a resemblance matrix calculated by multiple regression models. Additionally, a marginal test shows the amount of variation explained by each variable, ignoring the other variables, and a sequential test (forward direction) selects individual variables based on the Akaike information criterion (AIC). The lowest AIC values indicated the parsimonious model (the best combination of environmental variables that explain fish assemblages’ composition), and the proportion of explained variation attributed to each variable is added to the model as a function of the other variables already present. A distance-based redundancy analysis (dbRDA) was conducted based on the parsimonious model selected to visualize the relationship between environmental variables and years per zone.

## Results

**Fig 2:**
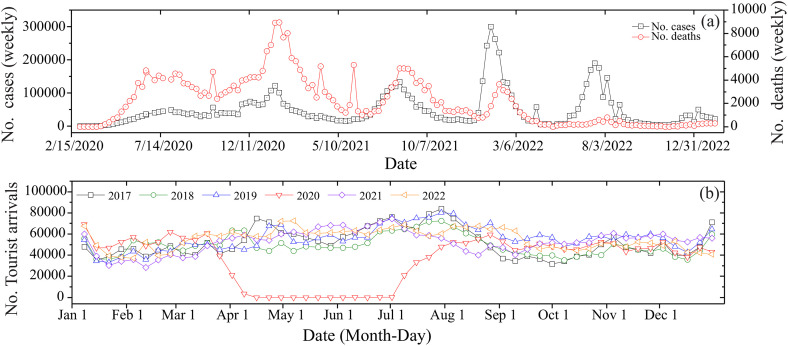
The number of COVID-19 cases and deaths recorded in Mexico (a) and the number of tourist arrivals at the city of Mazatlan (b).

The COVID-19 pandemic had a severe impact in Mexico, with significant mortality despite government containment efforts. Nationwide mobility restrictions were implemented only once, spanning April to July 2020 (Fig 2)—coinciding with the peak tourist season (Spring Break through summer holidays). Unlike the typical seasonal fluctuations observed in previous years, weekly visitor numbers stabilized at 40,000–80,000 during this period. The four multispectral algorithms demonstrated ecologically plausible patterns across the six coastal classes, with outputs consistent with known optical properties of coastal waters.

The two-way PERMANOVA indicated that differences existed in the four environmental parameters analyzed according to the coastal class, the year, and the interaction coastal class/year (coastal class pseudo-F_5,575_ = 22.4, p < 0.01; year pseudo-F3,575 = 11.9, p < 0.01; coastal class/year pseudo-F_15,575_ = 5.8, p < 0.01). Pairwise comparisons for the coastal class indicate that all zones were different among them according to the environmental parameters (S1 Table). With respect to the years, the analysis indicated that the post-pandemic years were not different from each other (2021 with 2022), indicating that the analyzed environmental factors were different between the pre-pandemic year, the pandemic year, and the post-pandemic period (S2 Table).

For the case of the pair-wise comparisons between coastal classes in the different years, in the touristic zone, the environmental factors were different between the pre-pandemic year to the post-pandemic period, and between the pandemic period to 2021, in the post-pandemic period (S3 Table). In the sewage zone, the environmental factors were different only between the pre-pandemic year with the year 2021, in the post-pandemic (S4 Table). In the harbor area, the environmental factors were different only during the pandemic with the year 2022, in the post-pandemic time (S5 Table). In the industrial zone, the environmental factors were not different during the pre-pandemic and the pandemic years but were different in all the other combinations (S6 Table). In the mangrove area, the environmental factors were different between the pandemic year with all the other years (S7 Table). Finally, in the coastline (preserved) area, pre-pandemic and pandemic years were different from the post-pandemic period (S8 Table).

**Fig 3:**
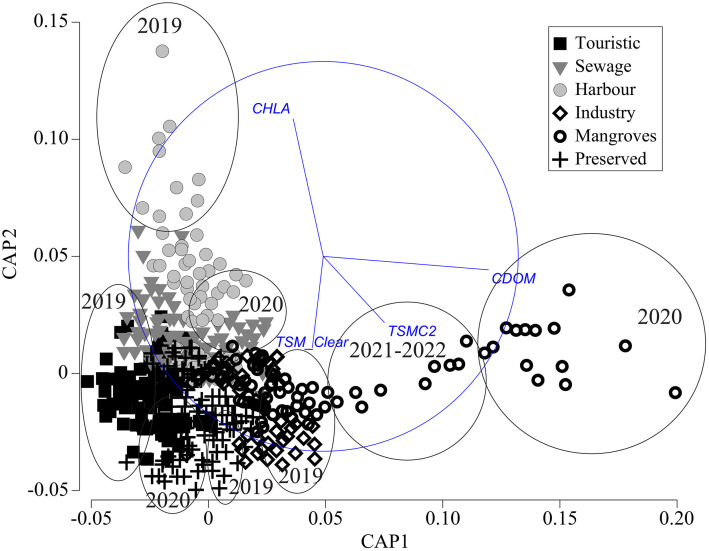
Canonical Analysis of Principal Coordinates (CAP) shows differences among the six coastal zones analyzed and years before, during, and after the COVID-19 lockdown.

The CAP, which merged all the coastal classes, revealed a pronounced temporal dichotomy, clearly separating pre-pandemic (2019), pandemic (2020), and post-pandemic recovery years (2021-2022) along the two ordination axes. CDOM concentrations were significantly higher in the mangrove zone during 2020 (Fig 3), while TSMC2 showed persistently high levels in this zone from 2021–2022. Spatial variability was particularly clear in 2019, with the mangrove and industrial zones exhibiting peak TSM_Clear concentrations, contrasting with maximum CHLA levels in the harbor zone. The pandemic year (2020) showed marked decreases in CHLA within sewage and harbor zones, coinciding with increases in both TSM_Clear and TSMC2—a pattern similarly observed in 2019 but restricted to sewage and touristic zones. Notably, preserved and touristic zones displayed elevated TSM_Clear and TSMC2 in 2020, while only TSM_Clear showed increases in the preserved zone during 2019.

**Fig 4:**
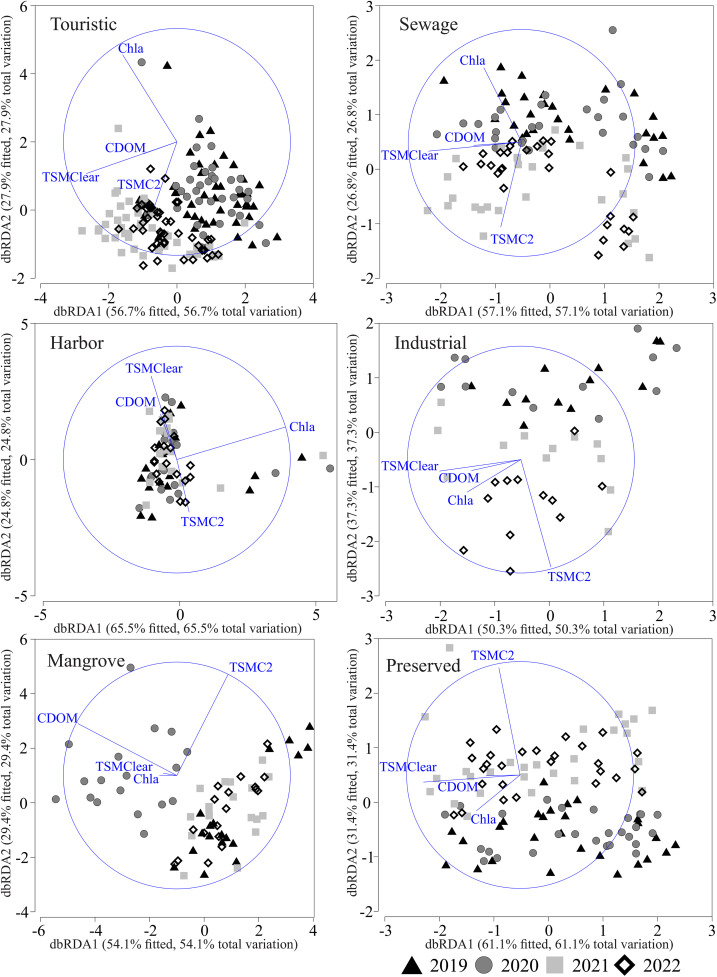
Distance-based redundancy analysis (dbRDA) plot performed from the distance-based linear model permutation test (DistLM) that describes the aggregation of sampling stations at every zone according to the year. Vectors indicate environmental variables with a significant effect, and markers correspond to the analyzed years.

The dbRDA revealed significant differences in water quality patterns across the coastal classes and years (Fig 4). The tourist zone showed particularly substantial changes, with water quality parameters following completely different trends before, during, and after the pandemic period. Specifically, CHLA levels remained consistently low across all four years, while TSMC2 underwent a noticeable increase during the post-lockdown phase. Even more intriguing was the seesaw pattern we observed with CDOM and TSM_Clear—these parameters decreased when human activity was restricted (2019–2020), only to bounce back sharply once normal conditions resumed (2021–2022). This reversal hints at some fundamental changes in the coastal ecosystem’s functioning during these extraordinary years. Industrial areas show a different pattern, with CDOM, CHLA, and TSM_Clear all dropping during the pandemic year before CHLA and TSMC2 staged a partial increase by 2022. Mangrove areas exhibited particularly strong interannual variability, with 2020 standing out for sharply increased CDOM, TSM_Clear, and CHLA, contrasting with elevated TSMC2 in 2019 and 2022. Preserved zones demonstrated rising CHLA during the pandemic year and increased TSMC2 post-pandemic, while select stations showed higher TSM_Clear and CDOM in 2021.

## Discussion

The abrupt cessation of global travel during 2020´s COVID-19 pandemic created an unplanned large-scale experiment in coastal ecosystem dynamics. Tourism-dependent regions experienced paradoxical outcomes—while economic analyses [1,12,17] revealed catastrophic losses, environmental monitoring [14,15] simultaneously recorded the most substantial improvement in water quality metrics seen in decades. This unique natural experiment offered a rare opportunity to quantify anthropogenic effects on coastal environments in popular tourist destinations.

Our study employed complementary statistical approaches to evaluate temporal and spatial variations in water quality indices. At the same time, Sentinel-2 satellite imagery has become suitable for coastal monitoring due to its unprecedented 10-m spatial resolution [11]—critical for studying small, dynamic systems like estuaries and coastal lagoons—we acknowledge its inherent limitations. While initially designated for terrestrial monitoring [6], since the Sentinel-2 coverage grid does not encompass the ocean, rather, it focuses exclusively on land, the sensor´s 5-day revisit capability, combined with freely available data, creates unique opportunities for aquatic time-series analysis. For this study, the Google Earth Engine [20] served as a critical processing platform, efficiently automating reflectance calculations at pixel scale across the entire study area while handling large amounts of multi-temporal data.

The mangrove zone, commonly viewed as an environmentally conserved area due to limited anthropogenic disturbances, displayed temporal variability in some water quality parameters. For instance, our results indicated that CDOM, TSM_Clear, and CHLA concentrations increased abruptly during the lockdown period, only to decrease in the following two years (2021−2022). This unexpected pattern may stem from two key factors: (i) continued wastewater discharges from exempted aquaculture operations during lockdowns, and (ii) natural seasonal processes like organic matter remineralization from mangrove leaf litter [29]. The trends we observed in the mangrove forest are consistent with those documented in an Iranian wetland during the COVID-19 lockdowns. Specifically, water indices data from the Sentinel-2 sensor indicated a 26% reduction in CHLA and a 16% reduction in turbidity, changes strongly linked to the quarantine’s reduction in human activity [30].

While tidal influences affect all coastal regions, the mangrove zone´s water quality dynamics diverge markedly from the adjacent industrial sector [31]. It appears that mangrove-derived nutrients—particularly from aboveground biomass—exert stronger control over the coastal lagoon´s biogeochemistry than inorganic nutrients from oceanic inputs. This influence is most clear in the system´s CHLA and CDOM cycles, where mangrove signatures dominate. Such findings highlight how these ecosystems function as biological filters, maintaining water quality in semi-enclosed coastal environments—a critical consideration for effective mangrove conservation strategies.

Analysis of water indices in the sewage and harbor coastal areas revealed no significant changes over the years, apart from a slight decrease in CHLA concentration. This stability is likely because both areas are affected by constant sewage discharges, even during the lockdown period, which had an estimated 500,000 permanent residents contributing wastewater. In the preserved zone, no significant variations were detected in CDOM levels over the years. This pattern was anticipated, as there are no apparent sources of organic matter pollution in this coastal region [32]. Only a few sampling stations near the Presidio River estuary showed an upsurge in CDOM levels, where the mangrove forest contributes organic matter [32]. The registered elevation in CHLA concentrations in the preserved zone during 2019 and 2020 aligned with a stronger coastal upwelling event, which brought inorganic nutrients to the surface layer and promoted phytoplankton bloom [33].

According to the data, there was a noticeable decrease in CDOM and TSM_Clear in the industrial zone during the year 2020 as compared to 2021 and 2022. Furthermore, there was a marked increase in TSMC2 in 2022. These observations suggest that the water clarity in the industrial zone was higher during the lockdown period in 2020, which could be attributed to the reduction in industrial activities during that time [34]. The levels of CDOM in the tourist zone experienced a significant decline in 2020. However, in the subsequent years of 2021 and 2022, there was an increase in both TSM_Clear and CDOM. On the other hand, the CHLA concentration remained consistently low across all years. These patterns are likely a result of the reduced clandestine discharges of organic matter from hotels into the coastal zone due to the absence of tourists [22].

According to Lock et al. [35], the TSM Case 2 water product is better suited for coastal waters with high land runoff and organic matter. However, a key drawback of this product is that the equations used are proprietary and can only be processed within the Sentinel Application Platform (SNAP) of the European Space Agency. Moreover, the CHLA index displayed similar patterns to the CDOM index in the majority of the coastal stations. This correlation is expected, as in coastal waters, the CHLA response is often linked to the input of organic matter from the coast [36].

Although a lockdown cannot alter the physical forces of the ocean, such as tides, our four-year study period highlighted the consistency of these background conditions. For instance, sea-surface elevation data based on geostrophic flow indicate that tropical storms and hurricanes in summer can increase sea level by approximately 35 cm in Mazatlan’s coastal zone [37]. Given the relatively short time series of our study, which did not encompass such events during the summer, the ocean’s influence remained relatively stable for all years. Furthermore, as the study focused on the April to June period—the driest months of the year—terrestrial influence from rainfall was negligible [38]. This stability in both oceanographic and terrestrial factors help to isolate the impact of the lockdown on water quality parameters.

While the water multispectral indices applied in our analysis were not originally developed for the specific optical conditions of our coastal study region, their consistent performance across all zones successfully revealed clear and coherent temporal trends. Thus, their utility for detecting coastal spatial-temporal changes was of utmost importance, even if local biophysical properties of water may limit absolute quantitative accuracy. The development of a regionally calibrated index, while a valuable future direction, was beyond the scope of our initial monitoring analysis.

## Conclusions

The study conducted on the multispectral water quality indices in the coastal zone of Mazatlan (Mexico) revealed that the year 2020, which was marked by confinement and limited human activity, showed a clear deviation from other years. Overall, the results reveal noticeable changes in remotely sensed water indices, demonstrating the effectiveness of Sentinel-2 for monitoring environmental responses. The industrial and tourist zones experienced a decline in CDOM, measuring 1.83 and 1.49 m^-1^, respectively, as well as in total suspended matter, which was 7.58 and 8.3 mg/L, respectively. This decline may be attributed to the absence of tourists and industrial activities. In contrast, the mangrove zone showed the highest CDOM level at 3.77 m^-1^, which was expected. The TSMC2, CDOM, CHLA, and TSM_Clear water indices demonstrated to be the most useful for detecting differences among the six coastal classes of this area. However, the study was limited by the lack of field data, particularly concerning organic water pollution variables like fecal coliform. Although spaceborne sensors were the only means of obtaining information and assessing the water quality, the results should be interpreted as relative changes in unverified proxies. We recommend testing the algorithms in other coastal areas using the freely accessible satellite data from the Sentinel-2 mission on the Copernicus server.

## Supporting information

S1 Table Pair-wise comparisons from PERMANOVA testing differences among the analyzed coastal classes. Italic and bold characters indicate significant differences (p-value < 0.05).
(DOCX)

S2 Table Pair-wise comparisons from PERMANOVA testing differences among the analyzed years. Italic and bold characters indicate significant differences (p-value < 0.05).
(DOCX)

S3 Table Pair-wise comparisons from PERMANOVA testing differences among the analyzed years in the touristic area. Italic and bold characters indicate significant differences (p-value < 0.05).
(DOCX)

S4 Table Pair-wise comparisons from PERMANOVA testing differences among the analyzed years in the sewage area. Italic and bold characters indicate significant differences (p-value < 0.05).
(DOCX)

S5 Table Pair-wise comparisons from PERMANOVA testing differences among the analyzed years in the harbor area. Italic and bold characters indicate significant differences (p-value < 0.05).
(DOCX)

S6 Table Pair-wise comparisons from PERMANOVA testing differences among the analyzed years in the industry area. Italic and bold characters indicate significant differences (p-value < 0.05).
(DOCX)

S7 Table Pair-wise comparisons from PERMANOVA testing differences among the analyzed years in the mangrove area. Italic and bold characters indicate significant differences (p-value < 0.05).
(DOCX)

S8 Table Pair-wise comparisons from PERMANOVA testing differences among the analyzed years in the coastline area. Italic and bold characters indicate significant differences (p-value < 0.05).
(DOCX)

S1 File Covid Mzt. (ZIP)
