# Supplementary material for: Assessing COVID-19 lockdown effects on coastal water quality in a strongly impacted tourist destination using Sentinel-2 multispectral data
Source: PLoS One. 2025 Oct 30;20(10):e0334974. doi: 10.1371/journal.pone.0334974 (PMC12574896; doi:10.1371/journal.pone.0334974)
Supplement: S1 Table — Italic and bold characters indicate significant differences (p-value < 0.05). (DOCX) [file pone.0334974.s001.docx]

**S1 Table. Pair-wise comparisons from PERMANOVA testing differences among the analyzed coastal classes.** Italic and bold characters indicate significant differences (p-value < 0.05).

| **Groups** | **t** | **p-value** | **permutations** |
| --- | --- | --- | --- |
| Touristic, sewage | 3.3068 | ***2e-5*** | 95388 |
| Touristic, harbor | 3.5083 | ***1e-5*** | 95382 |
| Touristic, industry | 3.494 | ***1e-5*** | 95307 |
| Touristic, mangroves | 8.093 | ***1e-5*** | 95330 |
| Touristic, coastline | 2.4023 | ***0.0029*** | 95274 |
| sewage, harbor | 2.2267 | ***0.0026*** | 95449 |
| sewage, industry | 4.2817 | ***1e-5*** | 95429 |
| sewage, mangroves | 7.0275 | ***1e-5*** | 95297 |
| sewage, coastline | 3.8708 | ***1e-5*** | 95334 |
| harbor, industry | 3.0348 | ***2e-5*** | 95480 |
| harbor, mangroves | 5.7057 | ***1e-5*** | 95277 |
| harbor, coastline | 4.0846 | ***1e-5*** | 95477 |
| industry, mangroves | 3.7229 | ***1e-5*** | 95331 |
| industry, coastline | 2.9602 | ***0.0003*** | 95227 |
| mangroves, coastline | 6.365 | ***1e-5*** | 95218 |
