# Supplementary material for: Assessing COVID-19 lockdown effects on coastal water quality in a strongly impacted tourist destination using Sentinel-2 multispectral data
Source: PLoS One. 2025 Oct 30;20(10):e0334974. doi: 10.1371/journal.pone.0334974 (PMC12574896; doi:10.1371/journal.pone.0334974)
Supplement: S2 Table — Italic and bold characters indicate significant differences (p-value < 0.05). (DOCX) [file pone.0334974.s002.docx]

**S2 Table. Pair-wise comparisons from PERMANOVA testing differences among the analyzed years.** Italic and bold characters indicate significant differences (p-value < 0.05).

| **Groups** | **t** | **p-value** | **permutations** |
| --- | --- | --- | --- |
| 2019, 2020 | 2.4844 | ***0.001*** | 95340 |
| 2019, 2021 | 3.5606 | ***0.001*** | 95337 |
| 2019, 2022 | 3.5013 | ***0.001*** | 95410 |
| 2020, 2021 | 3.0728 | ***0.001*** | 95235 |
| 2020, 2022 | 2.8628 | ***0.001*** | 95333 |
| 2021, 2022 | 0.60232 | 0.809 | 95339 |
