# Supplementary material for: Assessing COVID-19 lockdown effects on coastal water quality in a strongly impacted tourist destination using Sentinel-2 multispectral data
Source: PLoS One. 2025 Oct 30;20(10):e0334974. doi: 10.1371/journal.pone.0334974 (PMC12574896; doi:10.1371/journal.pone.0334974)
Supplement: S3 Table — Italic and bold characters indicate significant differences (p-value < 0.05). (DOCX) [file pone.0334974.s003.docx]

**S3 Table. Pair-wise comparisons from PERMANOVA testing differences among the analyzed years in the touristic area.** Italic and bold characters indicate significant differences (p-value < 0.05).

| **Groups** | **t** | **p-value** | **permutations** |
| --- | --- | --- | --- |
| 2019, 2020 | 1.2446 | 0.1986 | 9960 |
| 2019, 2021 | 3.0549 | ***0.0003*** | 9947 |
| 2019, 2022 | 2.4162 | ***0.0021*** | 9949 |
| 2020, 2021 | 1.8445 | ***0.0293*** | 9963 |
| 2020, 2022 | 1.0752 | 0.3219 | 9948 |
| 2021, 2022 | 1.2446 | 0.2009 | 9941 |
