# Supplementary material for: Assessing COVID-19 lockdown effects on coastal water quality in a strongly impacted tourist destination using Sentinel-2 multispectral data
Source: PLoS One. 2025 Oct 30;20(10):e0334974. doi: 10.1371/journal.pone.0334974 (PMC12574896; doi:10.1371/journal.pone.0334974)
Supplement: S4 Table — Italic and bold characters indicate significant differences (p-value < 0.05). (DOCX) [file pone.0334974.s004.docx]

S4 Table. Pair-wise comparisons from PERMANOVA testing differences among the analyzed years in the sewage area. Italic and bold characters indicate significant differences (p-value < 0.05).

| **Groups** | **t** | **p-value** | **permutations** |
| --- | --- | --- | --- |
| 2019, 2020 | 0.85756 | 0.5063 | 9942 |
| 2019, 2021 | 1.7857 | ***0.0351*** | 9939 |
| 2019, 2022 | 1.419 | 0.1091 | 9957 |
| 2020, 2021 | 1.6941 | 0.056 | 9955 |
| 2020, 2022 | 1.0749 | 0.304 | 9951 |
| 2021, 2022 | 0.90141 | 0.4184 | 9946 |
