# Supplementary material for: Assessing COVID-19 lockdown effects on coastal water quality in a strongly impacted tourist destination using Sentinel-2 multispectral data
Source: PLoS One. 2025 Oct 30;20(10):e0334974. doi: 10.1371/journal.pone.0334974 (PMC12574896; doi:10.1371/journal.pone.0334974)
Supplement: S5 Table — Italic and bold characters indicate significant differences (p-value < 0.05). (DOCX) [file pone.0334974.s005.docx]

S5 Table. Pair-wise comparisons from PERMANOVA testing differences among the analyzed years in the harbor area. Italic and bold characters indicate significant differences (p-value < 0.05).

| **Groups** | **t** | **p-value** | **permutations** |
| --- | --- | --- | --- |
| 2019, 2020 | 0.52805 | 0.8088 | 9955 |
| 2019, 2021 | 0.99735 | 0.3694 | 9938 |
| 2019, 2022 | 1.4136 | 0.1304 | 9949 |
| 2020, 2021 | 0.86992 | 0.4719 | 9938 |
| 2020, 2022 | 1.5886 | ***0.0457*** | 9951 |
| 2021, 2022 | 1.227 | 0.2701 | 9959 |
