# Supplementary material for: Assessing COVID-19 lockdown effects on coastal water quality in a strongly impacted tourist destination using Sentinel-2 multispectral data
Source: PLoS One. 2025 Oct 30;20(10):e0334974. doi: 10.1371/journal.pone.0334974 (PMC12574896; doi:10.1371/journal.pone.0334974)
Supplement: S6 Table — Italic and bold characters indicate significant differences (p-value < 0.05). (DOCX) [file pone.0334974.s006.docx]

S6 Table. Pair-wise comparisons from PERMANOVA testing differences among the analyzed years in the industry area. Italic and bold characters indicate significant differences (p-value < 0.05).

| **Groups** | **t** | **p-value** | **permutations** |
| --- | --- | --- | --- |
| 2019, 2020 | 1.5086 | 0.0983 | 9899 |
| 2019, 2021 | 1.78734 | ***0.0487*** | 9916 |
| 2019, 2022 | 1.9968 | ***0.0117*** | 9919 |
| 2020, 2021 | 1.8233 | ***0.0359*** | 9907 |
| 2020, 2022 | 3.2772 | ***0.0001*** | 9916 |
| 2021, 2022 | 1.7934 | ***0.0389*** | 9924 |
