# Supplementary material for: Assessing COVID-19 lockdown effects on coastal water quality in a strongly impacted tourist destination using Sentinel-2 multispectral data
Source: PLoS One. 2025 Oct 30;20(10):e0334974. doi: 10.1371/journal.pone.0334974 (PMC12574896; doi:10.1371/journal.pone.0334974)
Supplement: S7 Table — Italic and bold characters indicate significant differences (p-value < 0.05). (DOCX) [file pone.0334974.s007.docx]

S7 Table. Pair-wise comparisons from PERMANOVA testing differences among the analyzed years in the mangrove area. Italic and bold characters indicate significant differences (p-value < 0.05).

| **Groups** | **t** | **p-value** | **permutations** |
| --- | --- | --- | --- |
| 2019, 2020 | 5.2969 | **0.0001** | 9948 |
| 2019, 2021 | 0.97298 | 0.381 | 9938 |
| 2019, 2022 | 1.1136 | 0.2637 | 9943 |
| 2020, 2021 | 5.7553 | **0.0001** | 9963 |
| 2020, 2022 | 5.8397 | **0.0001** | 9952 |
| 2021, 2022 | 0.47769 | 0.8603 | 9954 |
