# Supplementary material for: Assessing COVID-19 lockdown effects on coastal water quality in a strongly impacted tourist destination using Sentinel-2 multispectral data
Source: PLoS One. 2025 Oct 30;20(10):e0334974. doi: 10.1371/journal.pone.0334974 (PMC12574896; doi:10.1371/journal.pone.0334974)
Supplement: S8 Table — Italic and bold characters indicate significant differences (p-value < 0.05). (DOCX) [file pone.0334974.s008.docx]

S8 Table. Pair-wise comparisons from PERMANOVA testing differences among the analyzed years in the coastline area. Italic and bold characters indicate significant differences (p-value < 0.05).

| **Groups** | **t** | **p-value** | **permutations** |
| --- | --- | --- | --- |
| 2019, 2020 | 1.2665 | 0.1858 | 9952 |
| 2019, 2021 | 3.2596 | ***0.0001*** | 9940 |
| 2019, 2022 | 2.1386 | ***0.0117*** | 9949 |
| 2020, 2021 | 1.9524 | ***0.0268*** | 9958 |
| 2020, 2022 | 1.8411 | ***0.0392*** | 9958 |
| 2021, 2022 | 1.6031 | 0.077 | 9939 |
